# Drug-resistance dynamics of *Staphylococcus aureus* between 2008 and 2014 at a tertiary teaching hospital, Jiangxi Province, China

**DOI:** 10.1186/s12879-016-2172-0

**Published:** 2017-01-25

**Authors:** Kaisen Chen, Yanfang Huang, Qiuyue Song, Chenhui Wu, Xiaowen Chen, Lingbing Zeng

**Affiliations:** 1 Department of Clinical Laboratory, The First Affiliated Hospital of Nanchang University, 17 Yongwaizhengjie, Nanchang, 330006 China; 2 The College of Public Health of Nanchang University, Nanchang, 330006 China

**Keywords:** *S. aureus*, Drug resistance, Trend, China

## Abstract

**Background:**

To understand the relationship between the *Staphylococcus aureus* infection rate and the reasonable usage of antibiotics, which will help in the effective control of MRSA infection.

**Methods:**

All data were obtained by the application of the nosocomial infection surveillance network. Drug resistance, departmental sources, and isolated sites as well as infection rate variations of *S. aureus* were analyzed in the 7-year period in key departments.

**Results:**

Between 2008 and 2014, 2525 strains of *S. aureus* isolates, mainly from sputum, skin/soft tissue, bloodstreams were collected from several hospital departments including respiratory, burn, brain surgery, orthopedics, ICU, and emergency. During these periods, the resistance rate of *S. aureus* to most drugs, including oxacillin, tetracycline, erythromycin, clindamycin, gentamicin, and ciprofloxacin, showed a tendency to decrease. The resistance to sulphamethoxazole/trimethoprim showed the opposite trend (*P* = 0.075) and there were no *S. aureus* strains resistant to linezolid and vancomycin. The MRSA infection rate was different across crucial hospital departments, with the burns department and ICU maintaining a high infection level. Over the 7-year period, both the brain surgery and the emergency departments had an expected upward trend (*P* < 0.05), while the orthopedic department showed a clear downward trend (*P* < 0.05) in MRSA infection rate.

**Conclusion:**

Hospitals should continue to maintain the current pattern of antibiotic administration, while more effective measures should be taken to reduce the high MRSA infection rate in some important hospital departments.

## Background

*Staphylococcus aureus* (*S. aureus*) is one of the most common clinical pathogens, causing nosocomial and community-acquired infections [1, 2]. Since methicillin-resistant *S. aureus* (MRSA) was first discovered, MRSA has now quickly become a predominant infectious pathogen with increasing global prevalence [3, 4]. Because of higher drug-resistance rate, MRSA is more difficult to treat, requires longer hospitalization periods, and results in higher morbidity and mortality than methicillin-susceptible *S. aureus* (MSSA) [5]. According to data from the U.S. Centers for Disease Control and Prevention (CDC), the number of severe MRSA infections in 2012 was 75,000, of which 9,700 resulted in death; hence, MRSA was renamed “super bug” [6]. In order to achieve better treatment and prognosis, it is paramount to find and adopt measures to control MRSA infection. In terms of pathogenicity, *S. aureus* infection is characterized by different virulence and drug resistance [7, 8]. While virulence is an inherent characteristic of each *S. aureus* strain, the degree of drug resistance is mainly affected by medical activities, such as prescribing habits, hospitalization periods, and hospital environment [9]. Studies have shown that changes in the bacteria environment was the main reason for the appearance of drug-resistant strains, and not the uneven distribution of infections across different hospitals or wards [10, 11]. In recent years, owing to the broad application of antibiotics in hospital as well as self-medication in communities in China, bacteria drug-resistance has reached very high levels [12]. This prompted the Chinese Health Ministry to issue guidelines for a better use of antimicrobial drugs in hospitals. It is therefore necessary to assess the effects of these guidelines in different hospitals, to understand whether these regulations are effective. Here, for the first time we report the trend of *S. aureus* infection rate in different key departments of a general tertiary-care teaching hospital after these regulations were put in place. These findings might provide some valuable information to local clinicians for selecting the appropriate antibiotic against *S. aureus*, and to health public departments offering more effective treatment measures.

## Methods

### Setting and study design

The hospital of Nanchang University is a large, general tertiary-care teaching hospital and regional medical center with 3,500 beds, located in the southeast region of China. The hospital includes almost all departments, such as burns, brain surgery, orthopedics, respiratory, pediatrics, gynecology and obstetrics, hematology and oncology, emergency room, and intensive care unit (ICU).

All records from the Hospital Information Warehouse, Clinical Microbiology Laboratory, and Pharmacy Department, were retrospectively collected at yearly intervals by two researchers, to obtain hospital-wide population-level data. The data include almost all bacteria strains and information on antimicrobial resistance. For some important pathogens, such as *S. aureus*, more detailed information, including different isolated rates and drug susceptibility in key departments, was present.

Recently, the Chinese Ministry of Health issued several documents to promote the correct usage of antibiotics in recent years. The aim of this study is to understand the relationship between the trend of isolated rate and antimicrobial resistance of *S. aureus* according to the current pattern of antibiotic administration.

### Microbiology data

Different strains of *S. aureus* were identified using the VITEK-2 automated system (bioMérieux Inc., France). The same system was used to measure susceptibility to the antibiotics routinely tested (penicillin G, oxacillin, gentamicin, ciprofloxacin, clindamycin, erythromycin, tetracycline, linezolid, vancomycin, and sulphamethoxazole/trimethoprim). The drug susceptibility results were compared to the newest standard of the Clinical and Laboratory Standards Institute (CLSI) [13]. Strains showing resistance to oxacillin were defined as MRSA, as described in the manufacturer’s instructions. All *S. aureus*-positive clinical specimens (sputum, skin/soft tissue, blood fluid, etc.) obtained from the hospital in the period between January 2008 and December 2014, were included in the analysis (identical strains derived from the same patient were excluded). The reference strains used for *S. aureus* were ATCC25923 and ATCC29213.

### Statistical analysis

Univariate analysis was used to determine the difference in drug resistance levels between MRSA and MSSA strains. A *chi*-square test was used to evaluate the difference in categorical variables. Variation analyses of *S. aureus* drug-resistant rate were performed using SPSS 17.0 (Statistical Product and Service *Solutions*, SPSS Inc., USA). Statistical significance was defined as *P* < 0.05.

## Results

### Distribution of the isolated *S. aureus* strains

During the 7-year period (January 2008-December 2014), a total of 2,525 strains of *S. aureus* were isolated from different patients admitted to various hospital wards. Among those, 967 (38.3%, 967/2525) were recovered from sputum, 713 (28.2%, 713/2525) from blood samples, 411 (16.3%, 411/2525) from skin/soft tissue, 249 (9.9%, 249/2525) from other sterile body fluids, and 185 from other organ. 1223 (48.4%, 1223/2525) isolated strains were classified as MRSA by system assay. The amount of isolated *S. aureus* strains differed across the departments; respiratory had 411(16.3%, 411/2525), burn 319 (12.6%, 319/2525), brain surgery 235 (9.3%, 235/2525), orthopedics 201 (8.0%, 201/2525), emergency 191 (7.6%, 191/2525), and ICU 168 (6.6%, 168/2525). The other departments including pediatrics, infectious, and hematology, had much lower levels of *S. aureus* strains.

### Trend of drug-resistance rate between 2008 and 2014

As shown in Table 1, amongst all the drugs tested, penicillin G was the one to which most strains showed resistance to (97.6% (205/210) in 2008 and 73.7% (469/636) in 2014), while erythromycin ranked second (81.9% (172/210) in 2008 and 54.7% (348/636) in 2014). Not many *S. aureus* isolates were resistant to sulphamethoxazole/trimethoprim (17.1% (359/2525) in 2008 and 23.6% (596/2525) in 2014). All isolates were sensitive to linezolid and vancomycin. Trend analysis showed that *S. aureus* resistance to oxacillin, gentamicin, ciprofloxacin, clindamycin, erythromycin, and tetracycline significantly decreased over time (*P* < 0.05) (Table 1). The percentage of strains resistant to penicillin G showed a decreasing tendency, although the observed change was not significant (*P* = 0.127). The percentage of strains resistant to sulphamethoxazole/trimethoprim fluctuated, with an overall increasing trend (*P* = 0.075).

**Table 1:** Trend of drug-resistance rate at the first affiliated hospital of Nanchang University, 2008-2014

Year (N)	Penicillin G	Oxacillin	Gentamicin	Ciprofloxacin	Clindamycin	Erythromycin	Linezolid	Vancomycin	Tetracycline	Cotrimoxazole
2008 (210)	97.6	69.5	60.5	64.3	75.2	81.9	0	0	69.5	17.1
2009 (259)	96.5	58.3	47.5	59.1	67.6	74.9	0	0	63.3	16.6
2010 (257)	93.4	50.2	50.6	59.1	63.8	68.1	0	0	59.9	19.1
2011 (273)	91.9	67.4	42.1	50.2	59.7	68.9	0	0	54.9	24.9
2012 (399)	91.2	49.6	39.1	52.4	52.1	66.9	0	0	38.3	25.1
2013 (491)	91.2	48.3	40.1	52.1	53 4	60 1	0	0	49.3	13.4
2014 (636)	73.7	42.1	30.8	31.8	32.5	54.7	0	0	34.0	23.6
Time series analysis model										
Slope (β)	−2.323	−9.507	−7.285	−7.909	−7.365	−7.127	…	…	−7.815	3.160
*P*-value	0.127	0.002	0.007	0.005	0.007	0.008	…	…	0.005	0.075
Trend	Decreasing	Decreasing	Decreasing	Decreasing	Decreasing	Decreasing	…	-…	Decreasing	Increasing

### Trend of drug resistance rate in key departments

Because doctors in different departments have different habits and criteria for antibiotic usage, it was necessary to determine the drug-resistance situation in each department. Hence, we selectively analyzed the drug-resistance trend in the respiratory, burns, brain surgery, orthopedics, and emergency departments as well as in the ICU (Table 2). As shown in Appendix 1, the resistance incidence to almost all selected antibiotics (except for linezolid and vancomycin) had a decreasing trend during the 7-year period. On the other hand, the resistance rate to sulphamethoxazole/trimethoprim slightly increased. Based on the results collected, we found that the resistance rate of MRSA was usually higher than that of MSSA (Table 3).

**Table 2:** Antimicrobial susceptibility trend of *S. Aureus* in key hospital departments, 2008–2014

Period (N)	Penicillin G	Oxacillin	Gentamicin	Ciprofloxacin	Clindamycin	Eiythromycin	Linezolid	Vancomycin	Tetracycline	Cotrimoxazole
Respiratory Dept (411)										
*X* ^2^ value	0.59	3.07	3.88	4.46	3.14	1.35	–	–	3.94	0.91
*P* value	0.44	0.08	0.05	0.04	0.08	0.24	–	–	0.05	0.34
Bum Dept (319)										
*X* ^2^ value	8.53	3.26	8.72	11.94	10.00	10.24	–	–	9.39	5.97
*P* value	0.00	0.07	0.00	0.00	0.00	0.00	–	–	0.00	0.02
Brain surgery (235)										
*X* ^2^ value	1.33	6.41	4.71	6.89	5.30	4.87	–	–	6.48	1.64
*P* value	0.25	0.01	0.03	0.01	0.02	0.03	–	–	0.01	0.20
Orthopedics (201)										
*X* ^2^ value	1.19	4.14	4.86	6.72	7.61	8.71	–	–	8.22	1.92
*P* value	0.28	0.04	0.03	0.01	0.01	0.00	–	–	0.00	0.17
Emergency Dept (191)										
*X* ^2^ value	3.13	6.97	5.68	6.86	5.88	5.48	–	–	5.05	2.44
*P* value	0.08	0.01	0.02	0.01	0.02	0.02	–	–	0.02	0.12
ICU (168)										
*X* ^2^ value	0.83	1.84	2.74	2.31	1.81	1.97	–	–	3.62	2.49
*P* value	0.36	0.18	0.10	0.13	0.18	0.16	–	–	0.06	0.12

**Table 3:** Antimicrobial susceptibility and isolated rates (%) of MRSA and MSSA in key departments

Period (N)	Penicillin G	Oxacillin	Gentamicin	Ciprofloxacin	Clindamycin	Erythromycin	Linezolid	Vancomycin	Tetracycline	Cotrimoxazole
Respiratory Dept (411)										
MRS A (190)	100.0	100.0	64.7	75.3	83.7	94.2	0	0	64.7	16.8
MSSA (221)	79.2	0.0	24.9	40.3	49.3	70.1	0	0	40.3	8.1
OR	0.79	–	0.38	0.23	0.59	0.74	–	–	0.62	0.49
95%CI	0.59–1.06	–	0.26–0.57	0.16–0.34	0.43–0.81	0.55–1.00	–	–	0.44–0.88	0.25–0.92
*P* value	0. 10	–	0.00	0.00	0.00	0.05	–	–	0.00	0.02
Bum Dept (319)										
MRS A (296)	100.0	100.0	48.6	55.4	56.8	188	0	0	47.0	26.7
MSSA (23)	34.8	0.0	17.4	56.5	47.8	52.2	0	0	52.2	21.7
OR	0.35	–	0.45	0.24	0.84	0.82	–	–	1.11	0.81
95%CI	0.13–0.82	–	0.11–1.36	0.04–0.80	0.36–1.86	0.36–1.77	–	–	0.49–2.41	0.23–2.29
*P* value	0.01	–	0.14	0.01	0.65	0.59	–	–	0.78	0.69
Brain surgery (235)										
MRSA (150)	100.0	100.0	50.7	84.7	80.7	80.7	0	0	88	38
MSSA (85)	85.9	0.0	25.9	25.9	30.6	43.5	0	0	27	5
OR	0.86	–	0.51	0.30	0.38	0.54	–	–	0.54	0.23
95%CI	0.57–1.29	–	0.28–0.90	0.17–0.53	0.22–0.64	0.33–0.87	–	–	0.31–0.92	0.07–0.62
*P* value	0.44	–	0.01	0.00	0.00	0.01	–	–	0.02	0.00
Orthopedics (201)										
MRSA (75)	100.0	100.0	93.3	86.7	64.0	65.3	0	0	42	27
MSSA (126)	85.4	0.0	29.4	41.3	41.3	44.4	0	0	54	13
OR	0.85	–	0.31	0.48	0.64	0.68	–	–	0.76	0.29
95%C1	0.55–1.31		0.19–0.53	0.29–0.78	0.38–1.08	0.41–1.13	–	–	0.45–1.30	0.13–0.62
*P* value	0.44	–	0.00	0.00	0.08	0.11	–	–	0.29	0.00
Emergency Dept (191)										
MRSA (90)	100.0	100.0	55.6	67.8	70.0	71.1	0	0	62	19
MSSA (101)	61.4	0.0	32.7	32.7	40.6	53.5	0	0	52	17
OR	0.61	–	0.59	0.48	0.58	0.75	–	–	0.75	0.80
95%CI	0.39–0.96	–	0.34–1.02	0.28–0.83	0.35–0.97	0.46–1.22	–	–	0.46–1.22	0.36–1 73
*P* value	0.03	–	0.05	0.01	0.03	0.22	–	–	0.22	0.53
ICU (168)										
MRSA (131)	100.0	100.0	39.7	79.4	92.4	83.2	0	0	83	51
MSSA (37)	81.1	0.0	18.9	45.9	27.0	43.2	0	0	14	4
OR	0.81	–	0.48	0.34	0.29	0.52	–	–	0.60	0.28
95%CI	0.45–1.44	–	0.17–1.18	0.14–0.74	0.12–0.63	0.26–1.02	–	–	0.28–1.22	0.07–0.84
*P* value	0.44	–	0.09	0.00	0.00	0.04	–	–	0.13	0.01

**Fig. 1:**
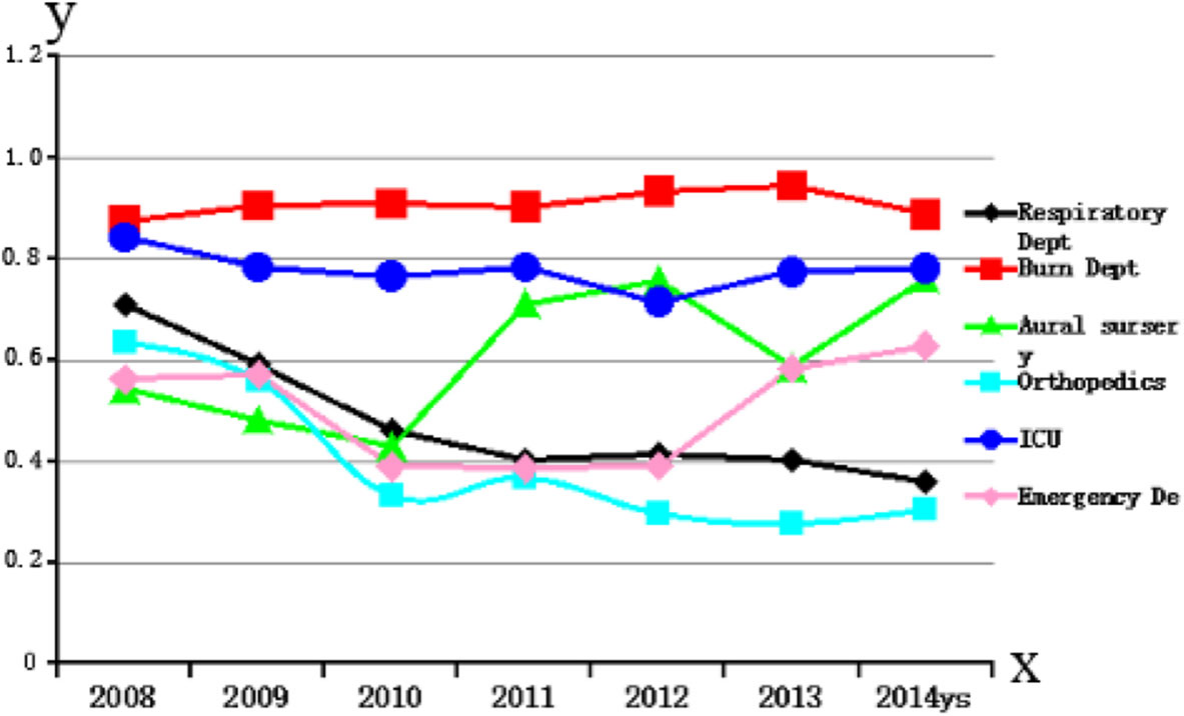
Dynamics of MRSA infection frequency in key departments of the first affiliated hospital of Nanchang University, 2008–2014. MRSA infection frequency (ratio) is presented on the y-axis

Because MRSA is an important pathogen and indicates a higher risk of contracting *S. aureus* infection [14], it was necessary to analyze the variation in MRSA infection frequency in key hospital departments during the same 7-year period. As shown in Fig. 1, MRSA infection was maintained at high levels in the burns department and ICU over the 7-year period; brain surgery and the emergency department showed an increased MRSA infection rate over time (*P* < 0.05), while both the respiratory and orthopedics departments had a decreasing trend, although the former did not attain statistical significance (*P* = 0.08 and 0.04, respectively).

**Fig. 2:**
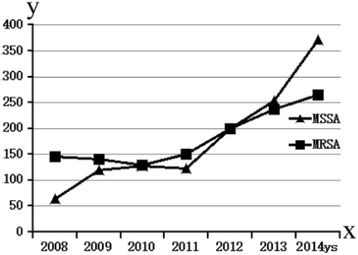
Frequency (no. of isolates) of MRSA and MSSA isolated at the first affiliated hospital of Nanchang University, China, 2008–2014

Additionally, the ratio of MRSA and MSSA isolates changed over time. Although the incidence of MRSA isolates was higher than that of MSSA in the period between 2008 and 2012, it decreased in the following years, reaching less than 40% in 2014 (Fig. 2).

## Discussion

As a major human pathogen, *S. aureus* causes a wide range of infections, such as lung disease, bacteremia, infectious endocarditis, as well as skin/soft tissue, and osteoarticular and device-related infections [15]. The epidemiological characteristics of *S. aureus*, especially MRSA, change rapidly. For example, although methicillin was adopted for the treatment of penicillin-resistant *S. aureus* infection in 1959, methicillin-resistant strains emerged immediately afterwards, becoming one of the most important strains worldwide during the following decades [16]. Furthermore, people with MRSA infection are 64.0% more likely to die than people with MSSA infection, hence increasing the cost of health care due to lengthier stays in hospital and more intensive care [17]. It is therefore necessary to adopt new measures to decrease the MRSA infection frequency. Kim et al. [18] studied the trend of MRSA infection, finding that a method for decreasing the frequency of infection is a more reasonable use of antibiotics. In the present study, we showed that the isolated rate of MRSA decreased over a period of 7 years, from 69.5% (146/210) in 2008 to 42.1% (268/2525) in 2014 (*P* < 0.05). Although the actual reasons for the decrease are not known, it is likely that this is owing to the improved measures for antibiotic administration (Appendix 2). Indeed, in 2008, the Chinese Ministry of Health issued the “Notice on the relevant issues concerning the clinical application management of antibiotics” document. Moreover, in 2009, the Ministry contacted experts to reexamine the antibiotic prescriptions in order to achieve a more reasonable antibiotic usage in some tertiary hospitals. As a result, the utilization rate of antibiotics improved in the following years. For example, according to statistics from the pharmacy department in our hospital, the percentage of prophylactic antibiotics used to prevent infections in surgical aseptic operations significantly decreased from 73.4% (11056/15063) in 2008 to 33.3% (1272/38195) in 2014, and the rate of reasonable use of antibiotics increased from 44.50% (6703/15063) to 90.85% (3470/38195). Subsequently, the rate of rational use of antibiotics has been maintained at more than 90.0%, in line with the national relevant requirements. The Ministry of Health issued the “Clinical application of antibacterial drugs Management method” in 2012 and developed special protocols to alter the current use of antimicrobial drugs, which provided guidance for standard antibiotic usage, resulting in an even more reasonable use of antibiotics. Our results were consistent with studies supporting the idea that a rational use of antibiotics, particularly broad-spectrum antibiotics, can effectively reduce *S. aureus* drug-resistance rate [19, 20].

Despite the fact that MRSA had a higher resistant rate than MSSA, the infectious situation varied in different departments. In order to propose a better differential treatment, it was necessary to determine *S. aureus* infection rate in different departments.

According to the data collected from important hospital departments, we found that the MRSA isolated rate in both the burns department and ICU was maintained above 80%. This was probably owing to the fact that most patients in these two departments are seriously ill and need effective treatments; hence, broad-spectrum antibiotics are usually prescribed [21]. For example, vancomycin, cefoperazone/sulbactam, and ketoconazole are usually prescribed to treat severe burn patients in hospitals. Antibiotic usage in ICU had no limitations. Moreover, the majority of patients in these departments have long-term hospitalization and are immune-compromised, which usually lead to persistent infection and an excessive use of antibiotics [22]. The MRSA infection frequency in the brain surgery and emergency departments increased in the 7-year period, opposite to the overall trend in other departments. The most plausible reason was that some departments, such as brain surgery, emergency, internal medicine, etc., had established independent ICU wards, owing to the expansion of the hospital (from a capacity of 1,600 beds to 3,500 beds) in 2012. The respiratory and especially the orthopedics department displayed a decreasing trend throughout the years, perhaps because of a more strict usage of antibiotics.

The results of our study cannot be generalized because the analysis of the small number of isolated strains in some departments does not reach statistical significance for some antibiotics tested. We also did not know the antibiotic usage of each individual patient. Therefore, we do not have enough data to prove the relationship between sensible antibiotic usage and decreased *S. aureus* drug-resistance. Since the antibiotic prescription habit was the only factor that changed, we believe that the more strict usage of antibiotics is responsible for the decrease in drug-resistant strains. Through the rational use of antibiotics, the frequency and drug-resistance rate of MRSA infection showed a downward trend.

In contrast, most gram-negative bacteria still show high levels of drug-resistance. For instance, *K. pneumoniae* showed a rising resistance trend, in contrast to what is reported in the literature [23]. The opinion that a more rational antibiotic usage could decrease resistance does not apply to gram-negative bacilli. Whether there are some other factors leading to the high rate of antibiotic resistance still needs to be analyzed.

## Conclusion

*S.aureus* is an important pathogen. The drug-resistance dynamics showed that reasonable usage of antibiotics has huge valuable for decreasing the MRSA infectious rate in a teaching hospital, China.

## Appendix 1

**Table 4:** The infectious rate and trend in *S.aureus* in key departments

Period (N)	Penici llin G	Oxa cillin	Genta micin	Ciprof loxacin	Clinda mycin	Erythro mycin	Line zolid	Vanco mycin	Tetracy cline	Cotrim oxazole
Respiratory Dept (411)										
2008(50)	100.0	70.0	60.0	76.0	84.0	94.0	0	0	82.0	10.0
2009(52)	98.1	59.6	51.9	71.2	75.0	84.6	0	0	63.5	9.6
2010(41)	95.1	46.3	53.7	63.4	75.6	90.2	0	0	53.7	19.5
2011(52)	92.3	32.7	44.2	55.8	65.4	84.6	0	0	46.2	13.5
2012(49)	91.8	14.3	38.8	53.1	53.1	83.7	0	0	42.9	10.2
2013(71)	84.5	40.8	39.4	43.7	62.0	76.1	0	0	49.3	9.9
2014(96)	75.0	35.4	30.2	51.0	54.2	70.0	0	0	37.5	13.5
*χ* ^2^ value	0.59	3.07	3.88	4.46	3.14	1.35	–	–	3.94	0.91
*P* value	0.44	0.08	0.05	0.04	0.08	0.24	–	–	0.05	0.34
Burn Dept (319)										
2008(11)	100.0	90.9	45.4	54.5	81.8	90.9	0	0	63.6	27.3
2009(26)	100.0	92.3	38.5	50.0	80.8	76.9	0	0	76.9	26.9
2010(31)	100.0	90.3	41.9	54.8	67.7	71.0	0	0	54.8	29.0
2011(43)	100.0	95.3	46.5	48.8	62.8	67.4	0	0	55.8	32.6
2012(45)	95.6	88.9	26.7	48.9	60.0	60.0	0	0	48.9	26.7
2013(73)	94.5	93.2	30.1	53.4	49.3	57.5	0	0	38.4	20.5
2014(96)	90.6	89.6	37.5	50.0	39.6	52.1	0	0	34.4	25.0
*χ* ^2^ value	8.53	3.26	8.72	11.94	10.00	10.24	–	–	9.39	5.97
*P* value	0.00	0.07	0.00	0.00	0.00	0.00	--	–	0.00	0.02
Aural surgery (235)										
2008 (27)	100.0	48.1	51.8	70.4	81.5	85.2	0	0	63.0	11.1
2009 (24)	95.8	54.2	66.7	70.8	79.2	79.2	0	0	58.3	12.5
2010 (22)	95.4	59.1	40.9	63.6	59.1	72.7	0	0	54.5	22.7
2011 (31)	96.8	64.5	35.5	64.5	61.3	67.7	0	0	51.6	19.3
2012 (37)	97.3	70.3	40.5	56.8	62.2	59.4	0	0	45.9	16.2
2013 (41)	92.7	70.7	36.6	61.0	56.1	61.0	0	0	43.9	22.0
2014 (54)	88.9	66.7	33.3	61.1	51.9	53.7	0	0	38.9	20.4
*χ* ^2^ value	1.33	6.41	4.71	6.89	5.30	4.87	–	–	6.48	1.64
*P* value	0.25	0.01	0.03	0.01	0.02	0.03	–	–	0.01	0.20
Orthopedics(201)										
2008 (23)	100.0	65.2	60.9	56.5	52.2	56.5	0	0	60.9	17.4
2009 (21)	95.2	52.4	47.6	47.6	42.9	47.6	0	0	47.6	14.3
2010(21)	90.5	38.1	52.4	66.7	71.4	61.9	0	0	52.4	28.6
2011 (26)	92.3	38.5	53.8	69.2	53.8	57.7	0	0	50.0	26.9
2012 (28)	89.3	25.0	35.7	57.1	39.3	42.9	0	0	42.9	17.9
2013 (35)	88.6	37.1	42.9	62.9	54.3	54.3	0	0	48.6	17.1
2014 (47)	83.0	30.0	27.7	51.0	44.7	48.9	0	0	40.4	19.1
*χ* ^2^ value	1.19	4.14	4.86	6.72	7.61	8.71	–	–	8.22	1.92
*P* value	0.28	0.04	0.03	0.01	0.01	0.00	–	–	0.00	0.17
Emergency Dept (191)										
2008 (20)	95.0	60.0	65.0	65.0	80.0	85.0	0	0	85.0	15.0
2009 (17)	94.1	52.9	52.9	52.9	70.6	76.5	0	0	64.7	17.6
2010 (22)	81.8	63.6	68.2	63.6	68.2	77.3	0	0	72.7	18.2
2011 (21)	81.0	57.1	57.1	57.1	61.9	71.4	0	0	81.0	23.8
2012 (24)	75.0	41.7	50.0	37.5	50.0	62.5	0	0	62.5	25.0
2013 (32)	75.0	62.5	40.6	46.9	46.9	50.0	0	0	53.1	18.8
2014 (55)	69.1	58.2	32.7	40.0	38.2	45.5	0	0	38.2	16.4
*χ* ^2^ value	3.13	6.97	5.68	6.86	5.88	5.48	–	–	5.05	2.44
*P* value	0.08	0.01	0.02	0.01	0.02	0.02	–	–	0.02	0.12
ICU(168)										
2008 (18)	100.0	88.9	50.0	83.3	88.9	88.9	0	0	83.3	38.9
2009(24)	100.0	75.0	29.2	75.0	70.8	79.2	0	0	75.0	33.3
2010 (16)	100.0	81.2	50.0	93.8	87.5	68.8	0	0	62.5	43.8
2011(21)	95.2	76.2	47.6	81.0	85.7	66.7	0	0	66.7	42.9
2012(17)	100.0	70.6	35.3	47.1	70.6	58.8	0	0	41.2	35.3
2013(28)	92.9	82.1	32.1	75.0	78.6	82.1	0	0	50.0	28.6
2014(44)	90.9	72.7	25.0	61.4	72.7	72.7	0	0	43.2	22.7
*χ* ^2^ value	0.83	1.84	2.74	2.31	1.81	1.97	–	–	3.62	2.49
*P* value	0.36	0.18	0.10	0.13	0.18	0.16	–	–	0.06	0.12

## Appendix 2

**Table 5:** Relative antibiotics usage regulation issued by Ministry of Health of China

Time	URL; Content; Key points
Jan-28 − 2008	http://www.nhfpc.gov.cn/zhuzhan/wsbmgz/201304/53cdeec6d8f4461583296ab59bf1fb93.shtml. Content: Notice on strengthening the price management of medical institutions to control the unreasonable growth of medical expenses. Key points: Regulate the behavior of medical services, and encourage the use of low-cost drugs.
Apr-15–200	http://www.nhfpc.gov.cn/zhuzhan/wsbmgz/201304/9a70b1ec336846f8b3858262c8ed6e29.shtml. Content: Notice on further strengthening the management of clinical application of antibacterial drugs. Key points: 1. Strengthen the management of perioperative antimicrobial prophylaxis and application; 2. To strengthen the management of clinical application of fluoroquinolones; 3. In strict accordance with the provisions of the management system for the classification of antimicrobial drugs, strengthen the management of clinical application of antibiotics; 4. To strengthen the guidance and supervision of clinical application of antibacterial drugs and strengthen the guidance and supervision of clinical application of antibacterial drugs
Jul-15–2008	http://www.nhfpc.gov.cn/zhuzhan/wsbmgz/201304/caa37dae8bde44bc9a42dd10aeb9ff43.shtml. Content: Notice on strengthening the work of hospital infection control in multi- drug resistant bacteria. Key points: 1. Management of nosocomial infection in multiple drug resistant bacteria; 2. To establish and improve the monitoring of multi-drug resistant bacteria; 3. Prevention and control of the spread of multi-drug resistant bacteria; 4. Strengthen the rational use of antibacterial drugs;5. Strengthen education and training of medical personnel.
Mar-25–2009	http://www.nhfpc.gov.cn/zhuzhan/wsbmgz/201304/0bf024050b7c4f229bc3e49f75f1e0da.shtml. Content: Notice on the relevant issues concerning the clinical application of antimicrobial agents. Key points: 1. In order to strictly control the surgical treatment of type I incision and to strengthen the management of prophylactic application of antibiotics in perioperative period; 2. Strict control of the clinical application of fluoroquinolones; 3. Strict implementation of the management system of antimicrobial drugs; 4. To strengthen the monitoring of clinical microbiology and bacterial resistance, establish the early warning mechanism for the clinical application of antibacterial drugs.
May-7 − 2013	http://www.nhfpc.gov.cn/yzygj/s3585u/201305/823b9d131ff4416ab7b41b2c4e1f0e83.shtml. Content: Notice on further development of the special rectification activities of national antimicrobial drugs. Key words : 1. To clarify the clinical application management responsibility system; 2. Investigation of clinical application of antimicrobial agents; 3. Establishment of a technical support system for clinical application of antibacterial drugs; 4. Strict implementation of the management system of antimicrobial drugs; 5. To establish a system for the selection and evaluation of antimicrobial agents, and to strengthen the management of antimicrobial agents; 6. Increase the clinical application of antimicrobial drugs related indicators control efforts; 7. Surveillance and evaluation of clinical application of antimicrobial agents; 8. Strengthen the detection and monitoring of bacterial resistance in clinical microbiology; 9. Strict management of drug prescribing authority and the qualification management of drug in the drug of the pharmacist; 10. Improve the management and reward system of antibacterial drugs, and seriously investigate and deal with the irrational use of antibacterial drugs; 11. Establish and improve the clinical application of antimicrobial agents and bacterial resistance monitoring network.

## Abbreviations

CDC: Centers for diseases control and prevention
CLSI: Clinical and Laboratory Standards Institute
ICU: Intensive care unit
MRSA: Methicillin-resistant *S. aureus*
MSSA: Methicillin-resistant *S.aureus*
